# Association of CD40 Gene Polymorphisms With Systemic Lupus Erythematosus and Rheumatoid Arthritis in a Chinese Han Population

**DOI:** 10.3389/fimmu.2021.642929

**Published:** 2021-04-22

**Authors:** Qi Huang, Wang-Dong Xu, Lin-Chong Su, Xiao-Yan Liu, An-Fang Huang

**Affiliations:** ^1^ Department of Evidence-Based Medicine, School of Public Health, Southwest Medical University, Luzhou, China; ^2^ Department of Rheumatology and Immunology, Minda Hospital of Hubei Minzu University, Enshi, China; ^3^ Department of Nutrition and Food Hygiene, School of Public Health, Southwest Medical University, Luzhou, China; ^4^ Department of Rheumatology and Immunology, Affiliated Hospital of Southwest Medical University, Luzhou, China

**Keywords:** CD40, polymorphism, systemic lupus erythematosus, rheumatoid arthritis, susceptibility, clinical features, disease activity parameters

## Abstract

Systemic lupus erythematosus (SLE) and rheumatoid arthritis (RA) are complex autoimmune diseases. CD40 participates in inflammatory response, and promotes fibroblast proliferation, leading to occurrence and progression of SLE, RA. This study explores CD40 gene polymorphisms in SLE and RA patients from a Chinese Han population. Two hundred SLE patients, 340 RA patients, and 900 healthy controls were enrolled. Genomic DNA was extracted from peripheral blood, and six polymorphisms of CD40 gene (rs3765456, rs1569723, rs73115010, rs13040307, rs1883832, and rs4810485) were detected by KASP method. Frequencies of rs1569723 genotypes AA, AC, AA+AC were significantly higher in RA patients as compared to those in healthy controls (P = 0.049, P = 0.024, P = 0.022). Frequencies of genotypes CT, CC+CT of rs1883832, and GT, GG+GT of rs4810485 were significantly higher in RA patients as compared to those in healthy controls (P = 0.012, P = 0.018, P = 0.009, P = 0.015). RA patients carrying rs13040307 C allele and rs73115010 T allele showed increased number of swollen joints. Moreover, frequency of allele T of rs13040307 was lower in SLE patients with positive anti-dsDNA and hematuria as compared to that in patients without these parameters (P = 0.038, P = 0.045). There were increased frequencies of genotype TT, allele T for rs13040307 and lower frequencies of genotype TT, allele T for rs73115010 in lupus patients with myositis (all P<0.05). Interestingly, frequencies of rs1569723 A allele, rs4810485 T allele were higher in SLE patients with myositis, and frequencies of rs3765456 A allele, rs1883832 T allele were lower in SLE patients with myositis (All P<0.05). In conclusion, CD40 gene polymorphisms may associate with susceptibility to SLE and RA.

## Introduction

Systemic lupus erythematosus (SLE) and rheumatoid arthritis (RA) are inflammatory autoimmune diseases, with features of chronic inflammation. The main characteristics of RA are progressive joints lesion, synovial hyperplasia, and production of auto-antibodies. SLE relates to various tissues and organs damage, such as skin, blood vessels, kidney (1, 2). Etiology of these two diseases remains unclear. It is recognized that gene polymorphisms have been involved in these disorders pathogenesis (3, 4). To date, at least 88 genomic regions have been identified associated with SLE susceptibility, such as TNFSF4, STAT4, and TNIP1, and about 106 susceptible genes were reported to associate with RA, such as CD28, IL3-CSF2, NFKBIE (5, 6). However, finding out more risk polymorphisms for RA and SLE will be helpful to better elucidate the pathogenesis of these diseases.

CD40 is a glycoprotein with molecular weight 45 to 50 kDa, consisting of 277 amino acids. It is a member of the tumor necrosis factor family of transmembrane glycoproteins and is expressed in different cells, such as B cells, monocytes, dendritic cells, endothelial and epithelial cells, smooth muscle cells, fibroblasts (7). CD40 is a potent T-cell costimulatory factor. It interacts with ligand CD40L, and plays an important role in adaptive immune response, for example, induction of Th1 cell response (8). Human CD40 gene is composed of eight introns and nine exons, located on chromosome 20q11.2 to 13.2. To date, association of CD40 gene and autoimmune disorders has been discussed widely. Chen et al. analyzed three polymorphisms (rs1883832, rs4810485, and rs1569723) of CD40 gene and risk of SLE in a Chinese population. They found that rs1883832 related to development of SLE (9). Nie et al. found relation between rs4810485 and SLE risk in a Chinese population (10). In European origins, studies showed that rs4810485 polymorphism was a risk factor for SLE, RA, and rs3765456 polymorphism was related to RA susceptibility in Korea population (11–13). However, association of rs73115010, rs13040307, and rs1569723 polymorphisms with SLE and RA were discussed in different ethnicities, and showed inconsistent conclusions. Therefore, to better clarify the relationship between CD40 gene polymorphisms and SLE, RA susceptibility, we conducted the present study in a Chinese Han population to evaluate six polymorphisms (rs3765456, rs1569723, rs73115010, rs13040307, rs1883832, and rs4810485) with relation to SLE and RA patients by co-dominant, dominant, and recessive genetic models.

## Materials and Methods

### Study Sample

This case-control study recruited 200 SLE patients (age, 38.0 [27.0–48.0] years), 340 RA patients (age, 56.0 [48.5–65.0] years) and 900 healthy controls (age, 38.0 [31.0–48.0] years) in Southern Chinese Han origin. All patients came from Department of Rheumatology and Immunology, Affiliated Hospital of Southwest Medical University. Healthy controls were from Physical Examination Center of Jiangyang District Center for Disease Control and Prevention in Luzhou city. RA and SLE patients were diagnosed according to the American College of Rheumatology and European League Against Rheumatism (ACR/EULAR) classification criteria (14, 15). RA patients disease activity was evaluated according to disease activity score 28 (DAS28) (16), and SLE patients disease activity was evaluated according to SLE disease activity index (SLEDAI) (17) (**Table 1**). This study was admitted by Ethic Research Committee of Southwest Medical University, and informed consent was collected from individual participant. Demographic and clinical characteristics of patients and controls were obtained as well. For instance, clinical characteristics myositis was diagnosed according to participants who had at least three of four items including clinical symptoms, serum creatine kinase level, electromyography, and muscle biopsy results (18).

**Table 1:** Characteristics of patients and controls.

Characteristics	SLE	RA	Healthy controls	P_1_	P_2_
Male (%)/female (%)	13.50/86.5	23.20/76.80	13.70/86.30	0.950	<0.001
Age (years)	38.0 (28.0-48.0)	57.0 (49.00–65.00)	38.0 (31.0-48.0)	0.059	<0.001
Tender joints (n)	–	12.00 (4.00–24.00)	–	–	–
Swollen joints (n)	–	7.50 (2.00–16.00)	–	–	–
ESR (mm/H)	–	60.00 (35.00–93.00)	–	–	–
CRP (mg/L)	–	20.70 (5.23–55.55)	–	–	–
Self-evaluation	–	74.00 (65.00–80.00)	–	–	–
HAQ score	–	20.00 (9.75–27.25)	–	–	–
IgG (g/L)	–	13.01 (9.60–16.20)	–	–	–
IgA (mg/L)	–	2.34 (1.78–3.20)	–	–	–
IgM (mg/L)	–	1.32 (0.97–2.03)	–	–	–
anti-CCP (U/ml)	–	59.00 (28.80–115.45)	–	–	–
C3 (g/L)	–	1.27 (1.11–1.43)	–	–	–
C4 (g/L)	–	0.27 (0.22–0.32)	–	–	–
RF (IU/ml)	–	128.40 (37.95–235.00)	–	–	–
DAS28	–	3.37 (5.31–7.53)	–	–	–
SLEDAI	5.63 (4.81–6.40)	–	–	–	–
Arthritis, n (%)	92 (46.00)	–	–	–	–
Discoid rash, n (%)	83 (41.50)	–	–	–	–
Alopecia, n (%)	50 (25.50)	–	–	–	–
Oral ulcers, n (%)	24 (12.00)	–	–	–	–
Vasculitis	18 (9.00)	–	–	–	–
Pleurisy, n (%)	17 (8.50)	–	–	–	–
Pericarditis, n (%)	16 (8.00)	–	–	–	–
Myositis, n (%)	11(5.50)	–	–	–	–
Fever, n (%)	38 (19.00)	–	–	–	–
Hypocomplementemia, n (%)	100 (50.00)	–	–	–	–
ds-DNA (+), n (%)	48 (24.00)	–	–	–	–
Thrombocytopenia, n (%)	29 (14.50)	–	–	–	–
Leukopenia, n (%)	24 (12.00)	–	–	–	–
Hematuria, n (%)	65 (32.50)	–	–	–	–
Proteinuria, n (%)	96 (48.00)	–	–	–	–
Pyuria, n (%)	22 (11.00)	–	–	–	–

SLE, systemic lupus erythematosus; RA, rheumatoid arthritis; ESR, erythrocyte sedimentation rate; CRP, C-reactive protein; HAQ, health assessment questionnaire; RF, rheumatoid factor; DAS28, disease activity score 28; SLEDAI, SLE disease activity index.

^1^Comparison between SLE and healthy controls.

^2^Comparison between RA and healthy controls.

### Single Nucleotide Polymorphism Selection

A systemic exploration for previous studies about CD40 gene polymorphism was carried out. According to the online tools: 1000 genomes project (https://www.ncbi.nlm.nih.gov/variation/tools/1000genomes/), all candidate single nucleotide polymorphisms (SNPs) must conform to three standard: pairwise tagging of HapMap population with r^2^≥0.8; a minor allele frequency (MAF)≥5%; Chinese Han Beijing (CHB) ethnicity. Finally, a total of six SNPs including rs3765456, rs1569723, rs73115010, rs13040307, rs1883832, and rs4810485 were selected.

### DNA Extraction and Genotyping

TIANamp Blood DNA kits (Tiangen, Beijing, China) were utilized for extracting genomic DNA. Genotyping of CD40 gene polymorphisms of these samples with qualified DNA concentration and purity was tested by Gene Company using KASP (Gene Company, Shanghai, China). KASP primers were summarized in **Supplementary Table 1**.

### Statistics

Statistical Package for the Social Science (SPSS) version 17.0 (SPSS Inc., Chicago) was used for statistical analysis. Categorical data were presented as frequency, percentage and analyzed by chi-square test. If continuous data was normally distributed, mean ± standard deviation (SD) was displayed, and independent samples t test was adopted. Otherwise, we chose median (interquartile range) and Wilcoxon ranks sum test for comparison. Odds ratio (OR) and 95% confidence interval (CI) were analyzed by logistic regression model. To evaluate deviation of each polymorphism, Hardy-Weinberg equilibrium test was used for SLE patients, RA patients, and healthy controls. There are three inheritance models, including co-dominant model, dominant model, and recessive model. For rs1883832, the co-dominant model includes CC versus TT, CT versus TT, the dominant model is CC+CT versus TT, and the recessive model is CC versus CT+TT. For rs1569723, the co-dominant model includes AA versus CC, AC versus CC, the dominant model is AA+AC versus CC, and the recessive model is AA versus AC+CC. For rs4810485, the co-dominant model includes GG versus TT, GT versus TT, the dominant model is GG+GT versus TT, and the recessive model is GG versus GT+TT. For rs13040307, the co-dominant model includes CC versus TT, CT versus TT, the dominant model is CC+CT versus TT, and the recessive model is CC versus CT+TT. For rs3765456, the co-dominant model includes GG versus AA, GA versus AA, the dominant model is GG+GA versus AA, and the recessive model is GG versus GA+AA. For rs73115010, the co-dominant model includes TT versus CC, TC versus CC, the dominant model is TT+TC versus CC, and the recessive model is TT versus TC+CC. Linkage disequilibrium (LD) and haplotype analysis were calculated by software: HaploView 4.2. A two-sided P value less than 0.05 was recognized as statistical significance.

## Results

### Characteristics of All Participants

There was no significant difference in gender (P = 0.950) and age (P = 0.059) between SLE patients and healthy controls. Compared RA patients with healthy controls, the age and gender was significantly different (both P<0.05), therefore, when we discussed differences between RA patients and healthy controls for the polymorphism, age and gender were adjusted. Other clinical characteristics were summarized in **Table 1**. All six polymorphisms were in accordance with Hardy-Weinberg equilibrium (**Supplementary Table 2**).

### Association Between CD40 Gene Polymorphisms and SLE

No differences for alleles and genotypes comparison of six SNPs between SLE patients and healthy controls were identified (**Table 2**). Subgroup analysis for association of CD40 gene polymorphisms and lupus patients by clinical features and laboratory parameters was carried out. Patients with positive anti-dsDNA had a lower frequency of rs13040307 allele T when compared to those with negative anti-dsDNA (P = 0.038). Patients with hematuria also showed a lower frequency of rs13040307 allele T as compared to that in patients without hematuria (P = 0.045). On the contrary, there was an increased frequency of genotype TT and allele T in lupus patients with myositis when compared to patients without the characteristic (P = 0.020, P = 0.002). A lower frequency of TT genotype and allele T for rs73115010 in lupus patients with myositis was obtained (P = 0.024, P = 0.004). Interestingly, higher frequencies of rs1569723 allele A, rs4810485 allele T and lower frequencies of rs3765456 allele A, rs1883832 allele T were noted in patients with myositis as compared to those in patients without the feature (P = 0.020, P = 0.030, P = 0.021, P = 0.032, respectively) (**Table 3** and **Supplementary Table 3**). SLEDAI was used to analyze disease activity for SLE patients, and association of genotypes for different polymorphisms and SLEDAI was evaluated. We found that none of the genotypes for the six polymorphisms associated with SLEDAI (**Supplementary Table 4**).

**Table 2:** Allele and genotype frequencies of six polymorphisms in the CD40 gene in SLE patients, RA patients and healthy controls.

SNP	Analysed model	SLE, n (%)	RA, n (%)	Controls, n (%)	Before adjustment^a^				After adjustment^a^			
					OR_1_ (95% CI)	P1	OR_2_ (95% CI)	P_2_	OR_1_ (95% CI)	P_1_	OR_2_ (95% CI)	P_2_
rs1883832	Gene types											
	CC	70 (45.0)	124 (36.5)	332 (36.9)	0.906 (0.568–1.447)	0.680	1.333 (0.900–1.974)	0.152	0.923 (0.578–1.476)	0.739	1.546 (0.964–2.479)	0.071
	CT	100 (50.0)	172 (50.6)	411 (45.7)	0.785 (0.502–1.229)	0.290	1.439 (1.022–2.181)	0.037	0.808 (0.515–1.266)	0.351	1.788 (1.135–2.817)	0.012
	TT	30 (15.0)	44 (12.9)	157 (17.4)	Reference		Reference		Reference		Reference	
	Allele											
	C	240 (60.0)	420 (61.8)	1075 (59.7)	1.012 (0.811–1.262)	0.918	1.089 (0.909–1.306)	0.354				
	T	160 (40.0)	260 (38.2)	725 (40.3)	Reference		Reference					
	Recessive model											
	CC	70 (45.0)	124 (36.5)	332 (36.9)	1.083 (0.786–1.494)	0.625	0.982 (0.758–1.273)	0.892	1.083 (0.786–1.494)	0.625	0.996 (0.726–1.366)	0.980
	CT+TT	130 (55.0)	216 (63.5)	568 (63.1)	Reference		Reference					
	Dominant model											
	CT+CC	170 (85.0)	296 (87.1)	743 (82.6)	0.835 (0.546–1.277)	0.465	1.422 (0.991–2.037)	0.055	0.855 (0.559–1.316)	0.473	1.681 (1.091–2.591)	0.018
	TT	30 (15.0)	44 (12.9)	157 (17.4)	Reference		Reference		Reference		Reference	
rs1569723	Gene types											
	AA	68 (34.0)	124 (36.5)	320 (35.6)	0.941 (0.594–1.492)	0.797	1.409 (0.951–2.087)	0.087	0.954 (0.601–1.515)	0.843	1.683 (1.002–2.571)	0.049
	AC	100 (50.0)	172 (50.6)	420 (46.6)	0.840 (0.542–1.301)	0.435	1.489 (1.021–2.173)	0.039	0.858 (0.553–1.332)	0.496	1.683 (1.072–2.642)	0.024
	CC	32 (16.0)	44 (12.9)	160 (17.8)	Reference		Reference		Reference		Reference	
	Allele											
	A	236 (59.0)	420 (61.8)	1060 (58.9)	1.005 (0.806–1.252)	0.967	1.128 (0.941-1.351)	0.193				
	C	164 (41.0)	260 (38.2)	740 (41.1)	Reference		Reference					
	Recessive model											
	AA	68 (34.0)	124 (36.5)	320 (35.6)	1.071 (0.776–1.479)	0.744	1.041 (0.803–1.349)	0.7764	1.069 (0.773–1.477)	0.680	1.083 (0.789–1.486)	0.623
	AC+CC	132 (66.0)	216 (63.5)	580 (64.4)	Reference		Reference		Reference		Reference	
	Dominant model											
	AC+AA	168 (84.0)	296 (87.1)	740 (82.2)	0.881(0.582–1.333)	0.607	1.453 (1.015–2.083)	0.040	0.898 (0.592–1.361)	0.610	1.650 (1.074–2.532)	0.022
	CC	32 (16.0)	44 (12.9)	160 (17.8)	Reference		Reference		Reference		Reference	
rs4810485	Gene types											
	GG	69 (34.5)	122 (35.9)	324 (36.0)	0.886 (0.554–1.416)	0.613	1.392 (0.937–2.069)	0.101	0.899 (0.562–1.438)	0.657	1.569 (0.975–2.523)	0.063
	GT	101 (50.5)	175 (51.5)	417 (46.3)	0.779 (0.498–1.218)	0.273	1.552 (1.061–2.270)	0.024	0.802 (0.512–1.255)	0.334	1.829 (1.160–2.884)	0.009
	TT	30 (15.0)	43 (12.6)	159 (17.7)	Reference		Reference		Reference		Reference	
	Allele											
	G	239 (59.7)	419 (61.6)	1065 (59.2)	1.024 (0.821–1.278)	0.830	1.108 (0.925–1.328)	0.267				
	T	161 (40.3)	261 (38.4)	735 (40.8)	Reference		Reference					
	Recessive model											
	GG	69 (34.5)	122 (35.9)	324 (36.0)	1.068 (0.774–1.473)	0.744	0.969 (0.767–1.290)	0.969	1.061 (0.768–1.465)	0.720	0.992 (0.723–1.362)	0.961
	GT+TT	131 (65.5)	218 (64.1)	576 (64.0)	Reference		Reference		Reference		Reference	
	Dominant model											
	GT+ GG	170 (85.0)	297 (87.4)	741 (82.3)	0.822(0.538–1.256)	0.408	1.481 (1.031–2.132)	0.033	0.894 (0.578–1.385)	0.427	1.715 (1.112–2.646)	0.015
	TT	30 (15.0)	43 (12.6)	159 (17.7)	Reference		Reference		Reference		Reference	
rs13040307	Gene types											
	CC	93 (46.5)	170 (50.0)	446 (49.5)	1.561(0.9652–2.527)	0.070	0.886 (0.580–1.354)	0.576	1.537 (0.948–2.492)	0.081	0.761 (0.445–1.301)	0.318
	CT	79 (39.5)	133 (39.1)	368 (40.9)	1.517 (0.928–2.478)	0.096	0.840 (0.545–1.296)	0.431	1.498 (0.915–2.451)	0.108	0.841 (0.487–1.451)	0.534
	TT	28 (14.0)	37 (10.9)	86 (9.6)	Reference		Reference		Reference		Reference	
	Allele											
	C	265 (66.3)	473 (69.6)	1260 (70.0)	0.841 (0.668–1.059)	0.141	0.979 (0.808–1.187)	0.831				
	T	135 (33.7)	207 (30.4)	540 (30.0)	Reference		Reference					
	Recessive model											
	CC	93 (46.5)	170 (50.0)	446 (49.5)	1.130 (0.831–1.536)	0.482	1.018 (0.793–1.306)	0.889	1.124 (0.826–1.529)	0.457	0.876 (0.647–1.186)	0.392
	CT+TT	107 (53.5)	170 (50.0)	454 (50.4)	Reference		Reference		Reference		Reference	
	Dominant model											
	CT+CC	172 (86.0)	303(89.1)	814 (90.4)	1.541(0.976–2.433)	0.072	0.865 (0.576–1.300)	0.486	1.520 (0.960–2.404)	0.074	0.796 (0.475–1.333)	0.387
	TT	28 (14.0)	37 (10.9)	86 (9.6)	Reference		Reference		Reference		Reference	
rs3765456	Gene types											
	GG	72 (36.0)	119 (35.0)	309 (34.3)	1.094 (0.708–1.690)	0.686	1.202 (0.818–1.767)	0.348	1.118 (0.722–1.730)	0.616	1.454 (0.913–2.315)	0.115
	GA	89 (44.5)	172 (50.6)	438 (48.7)	1.254 (0.825–1.907)	0.289	1.226 (0.850–1.770)	0.276	1.294 (0.849–1.973)	0.230	1.433 (0.921–2.229)	0.111
	AA	39 (19.5)	49 (14.4)	153 (17.0)	Reference		Reference		Reference		Reference	
	Allele											
	G	233 (58.3)	410 (60.3)	1056 (58.7)	0.983 (0.789–1.225)	0.878	1.070 (0.894–1.281)	0.462				
	A	167 (41.7)	270 (39.7)	744 (41.3)	Reference		Reference					
	Recessive model											
	GG	72 (36.0)	119 (35.0)	309 (34.3)	0.929 (0.675–1.280)	0.681	1.030 (0.793–1.338)	0.860	0.929 (0.670–1.280)	0.652	1.108 (0.805–1.525)	0.529
	GA+AA	128 (64.0)	221 (65.0)	591 (65.7)	Reference		Reference		Reference		Reference	
	Dominant model											
	GA+GG	161 (80.5)	291 (85.6)	747 (83.0)	1.182(0.800–1.748)	0.410	1.217 (0.896–1.724)	0.271	1.215 (0.820–1.799)	0.332	1.441 (0.947–2.193)	0.088
	AA	39 (19.5)	49 (14.4)	153 (17.0)	Reference		Reference		Reference		Reference	
rs73115010	Gene types											
	TT	89 (44.5)	155 (45.6)	423 (47.0)	1.451 (0.903–2.332)	0.124	0.941 (0.617–1.435)	0.777	1.430 (0.888–2.301)	0.141	0.808 (0.476–1.372)	0.430
	TC	82 (41.0)	148 (43.5)	382 (42.4)	1.422 (0.881–2.297)	0.150	0.995 (0.651–1.521)	0.981	1.415 (0.875–2.289)	0.157	1.055 (0.620–1.794)	0.844
	CC	29 (14.5)	37 (10.9)	95 (10.6)	Reference		Reference		Reference		Reference	
	Allele											
	T	260 (65.0)	458 (67.4)	1228 (68.2)	1.156 (0.920–1.452)	0.231	1.041 (0.862–1.256)	0.679				
	C	140 (35.0)	222 (32.6)	572 (31.8)	Reference		Reference					
	Recessive model											
	TT	89 (44.5)	155 (45.6)	423 (47.0)	1.106 (0.813–1.505)	0.532	0.945 (0.736–1.214)	0.657	1.094 (0.803–1.490)	0.569	0.773 (0.570–1.050)	0.099
	TC+CC	111 (55.5)	185 (54.4)	477 (53.0)	Reference		Reference		Reference		Reference	
	Dominant model											
	TC+TT	171 (85.5)	303 (89.1)	805 (89.4)	1.437(0.919–2.247)	0.137	0.966 (0.646–1.445)	0.868	1.422 (0.908–2.227)	0.123	0.919 (0.555–1.522)	0.743
	CC	29 (14.5)	37 (10.9)	95 (10.6)	Reference		Reference		Reference		Reference	

SNP, single-nucleotide polymorphism; SLE, systemic lupus erythematosus; RA, rheumatoid arthritis.

a Adjustment for age and sex; ^1^comparison between SLE and controls; ^2^comparison between RA and controls.

Description of alleles, genotypes of the six polymorphisms in SLE patients and healthy controls was listed as frequency, percentage. Differences of alleles, genotypes of the six polymorphisms between SLE patients and healthy controls were analyzed by chi-square test. Logistic regression calculated (adjusted) odds ratio and 95% confidence interval. Software SPSS 17.0 was used for the statistics.

**Table 3:** Analysis of CD40 gene polymorphisms (rs13040307, rs3765456, rs73115010) in SLE patients by clinical features.

Clinical features	rs13040307							rs3765456						rs73115010							
	Genotypefrequency (n)			P_1_	Allele frequency (n)		P_2_	Genotype frequency (n)			P_1_	Allele frequency (n)		P_2_	Genotype frequency (n)			P_1_	Allele frequency (n)		P_2_
	TT	TC	CC		T	C		AA	AG	GG		A	G		TT	TC	CC		T	C	
Arthritis																					
Positive	14	34	44	0.770	62	122	0.983	22	34	36	0.114	78	106	0.839	43	35	14	0.732	121	63	0.768
Negative	14	45	49		73	143		17	55	36		89	127		46	47	15		139	77	
Discoid rash																					
Positive	13	30	40	0.680	56	110	0.996	15	38	30	0.904	68	98	0.788	39	31	13	0.673	109	57	0.815
Negative	15	49	53		79	155		24	51	12		99	135		50	51	16		151	83	
Alopecia																					
Positive	7	21	22	0.907	35	65	0.760	10	22	18	0.994	42	58	0.953	22	20	8	0.949	64	36	0.809
Negative	28	58	71		100	200		29	67	54		125	175		67	62	21		196	104	
Oral ulcers																					
Positive	4	8	12	0.789	16	32	0.948	8	8	8	0.174	24	24	0.217	13	8	3	0.610	34	14	0.366
Negative	24	71	81		119	233		31	81	94		143	209		76	74	26		226	126	
Pleurisy																					
Positive	3	6	8	0.878	12	22	0.842	3	7	7	0.897	13	21	0.664	7	7	3	0.942	21	13	0.679
Negative	25	73	85		123	243		36	82	65		154	212		82	75	26		239	127	
Pericarditis																					
Positive	3	7	8	0.718	13	19	0.391	2	5	9	0.212	9	23	0.103	5	8	3	0.558	18	14	0.279
Negative	25	72	87		122	246		37	84	63		158	210		84	74	26		242	126	
Fever																					
Positive	7	13	18	0.608	27	49	0.716	8	15	15	0.786	31	45	0.850	16	15	7	0.778	47	29	0.593
Negative	21	66	75		108	216		31	74	57		136	188		73	67	22		213	111	
Hypocomplementemia																			
Positive	14	38	48	0.938	66	134	0.751	23	39	38	0.242	85	115	0.761	47	38	15	0.705	132	68	0.675
Negative	14	41	45		69	131		16	50	34		82	118		42	44	14		128	72	
ds-DNA(+)																					
Positive	5	14	29	0.086	24	72	0.038	12	23	13	0.281	47	49	0.100	26	15	7	0.244	67	29	0.259
Negative	23	65	64		111	193		27	66	59		120	184		63	67	22		193	111	
Thrombocytopenia																					
Positive	4	12	13	0.974	20	38	0.898	6	12	11	0.935	24	34	0.951	13	12	4	0.886	38	20	0.929
Negative	24	67	80		115	227		33	77	61		143	199		76	70	25		222	120	
Leukopenia																					
Positive	3	7	14	0.449	13	35	0.298	7	9	8	0.436	23	25	0.356	13	8	3	0.610	34	14	0.366
Negative	25	72	79		122	230		32	80	64		144	208		76	74	26		226	126	
Hematuria																					
Positive	8	19	38	0.057	35	95	0.045	17	25	23	0.225	59	71	0.306	34	23	8	0.310	91	39	0.146
Negative	20	60	55		100	170		22	64	49		108	162		55	59	21		169	101	
Proteinuria																					
Positive	17	34	45	0.273	68	124	0.498	21	38	37	0.393	80	112	0.974	44	35	17	0.336	123	69	0.706
Negative	11	45	48		67	141		18	51	35		87	121		45	47	12		137	71	
Pyuria																					
Positive	1	9	12	0.380	11	33	0.193	7	7	8	0.244	21	23	0.394	12	9	1	0.310	33	11	0.140
Negative	27	70	81		124	232		32	82	64		146	210		77	73	28		227	129	
Vasculitis																					
Positive	2	7	9	0.918	11	25	0.671	3	9	6	0.880	15	21	0.992	9	7	2	0.847	25	11	0.558
Negative	26	72	84		124	240		36	80	66		152	212		80	75	27		235	129	
Myositis																					
Positive	4	6	1	0.020	14	8	0.002	0	4	7	0.086	4	18	0.021	1	6	4	0.024	8	14	0.004
Negative	24	73	92		121	257		39	85	65		163	215		88	76	25		252	126	

P_1_, Patients positive versus patients negative using 3×2 contingency table.

P_2_, Patients positive versus patients negative using 2×2 contingency table.

### Association Between CD40 Gene Polymorphisms and RA

Differences of alleles and genotypes of CD40 gene polymorphisms between RA patients and healthy controls were shown in **Table 2**. Frequencies of genotypes AA, AC, and AA+AC of rs1569723 were different between RA patients and healthy controls after age and gender adjustment (AA vs CC, OR, 1.683; 95% CI, 1.002–2.571, P = 0.049; AC vs CC, OR, 1.683; 95% CI, 1.072–2.642, P = 0.024; AA+AC vs CC, OR, 1.650; 95% CI, 1.074–2.534; P = 0.022). Frequencies of genotypes CT, CT+CC of rs1883832 were different between RA patients and healthy controls after age and gender adjustment (CT vs TT; OR, 1.788; 95% CI, 1.135–2.817; P=0.012; CT+CC vs TT: OR, 1.681; 95% CI, 1.091–2.591; P = 0.018). For rs4810485, statistical significance was obtained for comparison of GT with TT, and GT+GG with TT after adjustment by age and gender (GT vs TT: OR, 1.829; 95% CI, 1.160–2.884; P = 0.009; GT+GG vs TT: OR, 1.715; 95% CI, 1.112–2.646; P=0.015). The other three polymorphisms were not related to RA risk.

Association of disease activity in RA patients with CD40 gene polymorphisms was displayed in **Table 4**. For rs13040307, number of the swollen joints between patients with CC+CT genotype and CC genotype was different (P = 0.010). RA patients carrying rs73115010 TT+TC genotype had higher number of swollen joints as compared to the patients carrying CC genotype (P = 0.038). Other polymorphisms were not related to RA clinical parameters (**Supplementary Table 5**, **Supplementary Table 6**).

**Table 4:** Association of disease activity parameters in RA patients with CD40 gene polymorphisms (rs1569723, rs13040307).

Characteristics	rs13040307 [Median (P_25_–P_75_)]			P_1_	rs73115010 [Median (P_25_–P_75_)]			P_2_
	CC	CT	TT		TT	TC	CC	
Tender joints (n)	11.00 (4.00–23.75)	13.00 (5.00–24.00)	10.00 (4.00–26.00)	0.547	12.00 (4.00–23.25)	12.50 (5.00–27.00)	10.00 (2.50–23.75)	0.438
Swollen joints (n)	5.50 (1.00–16.00)	10.00 (2.00–16.00)	6.00 (1.00–14.00)	0.010	7.00 (2.00–14.00)	10.00 (2.00–20.00)	4.00 (0.00–12.00)	0.038
ESR (n)	63.50 (39.00–89.50)	62.00 (35.00–92.00)	52.00 (29.00–96.00)	0.798	60.00 (34.00–95.00)	60.00 (37.25–87.45)	67.50 (26.50–102.78)	0.635
CRP (mg/L)	12.60 (6.36–44.30)	24.70 (5.15–62.99)	18.10 (5.40–51.30)	0.792	18.90 (5.30–55.40)	20.95 (4.50–57.35)	26.00 (6.40–63.85)	0.631
Self-evaluation	79.00 (68.75–83.50)	70.00 (65.0–80.00)	75.00 (65.00–85.00)	0.806	75.00 (65.00–80.00)	70.00 (65.00–80.00)	75.00 (65.00–89.00)	0.416
HAQ score	20.00 (7.00–28.00)	20.00 (10.00–27.00)	20.00 (12.00–27.75)	0.906	20.00 (10.00–28.25)	20.00 (9.00–26.00)	20.00 (8.50–28.00)	0.753
IgG (g/L)	12.50 (11.34–16.64)	12.95 (9.41–16.35)	13.18 (9.58–16.27)	0.807	13.04 (9.41–18.19)	12.95 (9.72–16.49)	13.34 (9.53–15.71)	0.807
IgA (mg/L)	2.69 (2.08–4.58)	2.28 (1.75–3.12)	2.38 (1.78–3.20)	0.298	2.59 (1.79–3.76)	2.32 (1.78–3.01)	1.98 (1.74–2.75)	0.298
IgM (mg/L)	1.41 (1.05–1.86)	1.38 (0.99–2.01)	1.24 (0.91–2.07)	0.775	1.34 (0.97–1.98)	1.29 (1.00–2.05)	1.51 (0.80–2.07)	0.775
RF (IU/L)	110.20 (29.38–216.25)	122.65 (33.60–268.80)	139.00 (41.10–236.00)	0.683	140.85 (45.23–318.38)	121.00 (32.33–226.25)	97.60 (31.00–236.00)	0.613
anti-CCP (U/mL)	59.80 (25.40–91.00)	58.40 (30.20–139.90)	60.35 (29.10–121.35)	0.748	59.40 (26.53–107.88)	57.80 (30.60–149.00)	52.50 (25.53–137.00)	0.713
C3 (g/L)	1.25 (1.15–1.46)	1.22 (1.09–1.45)	1.28 (1.15–1.38)	0.644	1.28 (1.10–1.46)	1.24 (1.12–1.37)	1.28 (1.16–1.43)	0.644
C4 (g/L)	0.28 (0.23–0.37)	0.27 (0.22–0.32)	0.26 (0.21–0.36)	0.518	0.26 (0.21–0.34)	0.27 (0.22–0.31)	0.29 (0.19–0.37)	0.518
DAS28	6.38 (5.19–7.46)	6.50 (5.36–7.67)	6.06 (5.17–7.42)	0.694	6.27 (5.06–7.42)	6.50 (5.44–7.67)	6.06 (5.17–7.42)	0.679

Characteristics	rs4810485 [Median (P_25_-P_75_)]				rs13040307 [Median (P_25_-P_75_)]			
	GG	TG	TT	P_1_	CC	TC	TT	P_2_
Tender joints (n)	12.00 (3.00–23.50)	11.00 (5.00–27.00)	12.0 (4.00–24.00)	0.727	11.00 (4.00–23.75)	13.00 (5.00–24.00)	10.00 (4.00–26.00)	0.547
Swollen joints (n)	4.00 (0.00–12.00)	10.00 (2.00–20.00)	7.00 (2.00–16.00)	0.498	5.50 (1.00–16.00)	10.00 (2.00–16.00)	6.00 (1.00–14.00)	0.010
ESR (n)	55.50 (26.50–100.50)	60.00 (31.5–87.00)	61.00 (36.00–95.00)	0.761	63.50 (39.00–89.50)	62.00 (35.00–92.00)	52.00 (29.00–96.00)	0.798
CRP (mg/L)	26.00 (6.40–54.35)	19.90 (4.21–50.00)	21.05 (5.50–58.00)	0.479	12.60 (6.36–44.30)	24.70 (5.15–62.99)	18.10 (5.40–51.30)	0.792
Self-evaluation	70.00 (62.50–85.00)	75.00 (65.00–85.00)	72.50 (66.50–80.00)	0.300	79.00 (68.75–83.50)	70.00 (65.0–80.00)	75.00 (65.00–85.00)	0.806
HAQ score	20.00 (11.50–26.50)	20.00 (8.25–27.00)	20.00 (10.00–28.00)	0.458	20.00 7.00–28.00)	20.00 (10.00–27.00)	20.00 (12.00–27.75)	0.906
IgG (g/L)	13.34 (9.53–15.71)	12.95 (9.84–16.40)	13.04 (9.31–16.30)	0.688	12.50 (11.34–16.64)	12.95 (9.41–16.35)	13.18 (9.58–16.27)	0.807
IgA (mg/L)	1.98 (1.74–2.75)	2.40 (1.79–3.01)	2.34 (1.67–3.64)	0.140	2.69 (2.08–4.58)	2.28 (1.75–3.12)	2.38 (1.78–3.20)	0.298
IgM (mg/L)	1.51 (0.80–2.07)	1.27 (0.96–2.09)	1.36 (0.99–1.95)	0.632	1.41 (1.05–1.86)	1.38 (0.99–2.01)	1.24 (0.91–2.07)	0.775
RF (IU/L)	91.45 (34.98–268.78)	131.35 (36.67–232.25)	129.10 (38.15–258.95)	0.540	110.20 (29.38–216.25)	122.65 (33.60–268.80)	139.00 (41.10–236.00)	0.683
anti-CCP (U/mL)	63.00 (27.2–99.00)	58.40 (31.50–148.15)	58.75 (25.93–111.38)	0.577	59.80 (25.40–91.00)	58.40 (30.20–139.90)	60.35 (29.10–121.35)	0.748
C3 (g/L)	1.28 (1.16–1.43)	1.23 (1.12–1.37)	1.28 (1.10–1.46)	0.718	1.25 (1.15–1.46)	1.22 (1.09–1.45)	1.28 (1.15–1.38)	0.644
C4 (g/L)	0.29 (0.19–0.37)	0.27 (0.22–0.31)	0.26 (0.21–0.34)	0.330	0.28 (0.23–0.37)	0.27 (0.22–0.32)	0.26 (0.21–0.36)	0.518
DAS-28	6.14 (5.00–7.52)	6.49 (5.47–7.54)	6.70 (5.16–7.57)	0.963	6.38 (5.19–7.46)	6.50 (5.36–7.67)	6.06 (5.17–7.42)	0.694

ESR, erythrocyte sedimentation rate; CRP, C-reactive protein; HAQ, health assessment questionnaire; RF, rheumatoid factor; DAS28, disease activity score 28.

^1^CC+CT versus TT genotype for rs13040307; ^2^TT+TC versus CC genotype for rs73115010.

### CD40 Haplotype Analysis for RA and SLE

**Figure 1:**
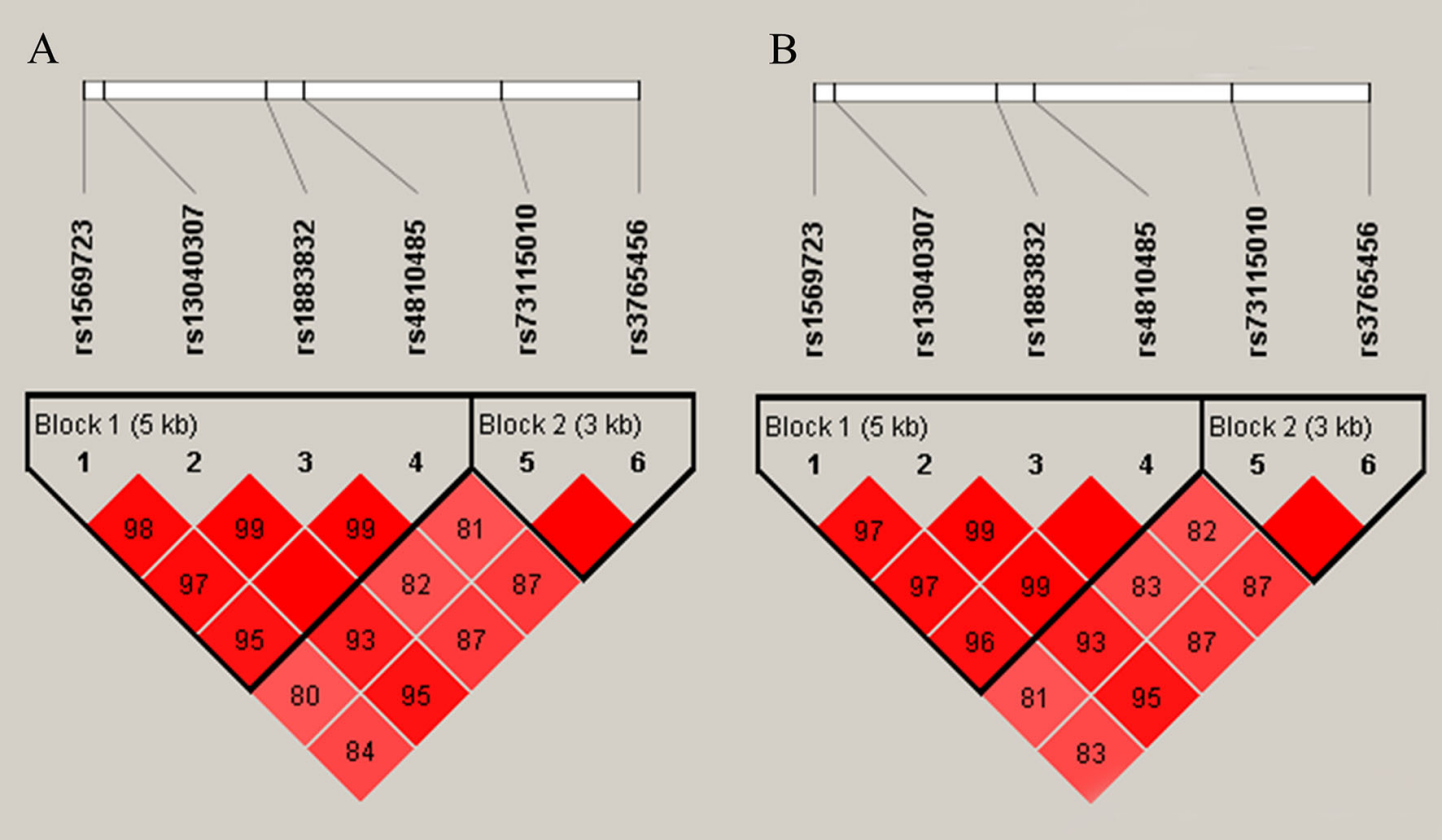
Linkage disequilibrium(LD) of six single nucleotide polymorphisms. The intensity of linkage disequilibrium (LD) is reflected in the color and numeric value (D′) of each box. Bright red means D′ ≥0.95, LOD ≥2. Shade of red means D′<0.95, LOD≥2. **(A)** LD of RA patients and controls. **(B)** LD of SLE patients and controls. Both of them show that block 1 consists of four polymorphisms including rs1569723 A/C, rs13040307 T/C, rs1883832 T/C, and rs4810485 T/G, block 2 consists of rs3765456 A/G and rs73115010 T/C.

Considering linkage disequilibrium of genes, we performed haplotype analysis for CD40 gene polymorphisms. Two blocks were defined by 95% CI (19). The first one consists of four polymorphisms including rs1569723, rs13040307, rs1883832, and rs4810485, and the other one consists of rs3765456 and rs73115010 both for RA and SLE (**Figures 1A, B**). Results showed that there was no significant difference between patients and controls regarding different haplotypes (**Supplementary Table 7**).

## Discussion

This study explored association of CD40 gene polymorphisms with susceptibility to SLE and RA in a Southern Chinese Han population. A total of six polymorphisms, rs3765456, rs1569723, rs73115010, rs13040307, rs1883832, and rs4810485 were recruited. For RA, the co-dominant model and dominant model of rs1569723, rs1883832, and rs4810485 polymorphisms associated with increased susceptibility to RA. Number of swollen joints were increased in RA patients with genotypes CC+TC of rs13040307 and TT+TC of rs373115010. For SLE, rs13040307 of CD40 gene was related to clinic features, including hematuria and anti-dsDNA. Interestingly, the six polymorphisms may be related to SLE patients with myositis.

SLE and RA are autoimmune disorders with genetic pathogenesis. For rs3765456, this polymorphism may be a risk factor for SLE susceptibility and associated with disease activity in Korea population (12), whereas this polymorphism was not significantly related to SLE in Northern Chinese population (10). In our study, there was no significant difference regarding this polymorphism and SLE risk in Southern Chinese Han population. Interestingly, SLE patients with myositis associated with a lower frequency of rs3765456 allele A, suggesting that rs3765456 allele A may affect the patients complicated with this feature. To date, no study has discussed association of rs3765456 polymorphism with RA risk. In this study, we found that this polymorphism was not related to RA risk. For rs1569723, a previous genome-wide association study reported that there was relation between RA and rs1569732 in European population (20). In our study, we confirmed that this polymorphism related to Chinese RA patients. With respect to relationship between rs1569732 and SLE, a study in Chinese population showed that there was significant difference between rs1569723 and SLE (9). The present study did not find significant association of this polymorphism and SLE risk in our Southern Chinese Han population. However, higher frequency of rs1569723 allele A was related to SLE patients with myositis in this study. Regarding rs13040307 and rs73115010, there were limited studies evaluating association of the two polymorphisms with disease susceptibility, including lupus or arthritis. Wu et al. reported that these two polymorphisms may not associate with SLE in Chinese population by a relatively small sample size (21). By contrast, a study in Korea population indicated that rs73115010 may increase the risk of SLE (12). In this study, there was no significant association of these polymorphisms and SLE, RA risk in our Southern Chinese Han population. Interestingly, rs13040307 polymorphism was related to disease activity parameters of SLE, including anti-dsDNA, hematuria, and myositis. In addition, RA patients carrying genotype CC+CT of rs13040307 and genotype TT+TC of rs73115010 had higher number of swollen joints. However, further studies with larger sample size and different ethnicities are needed to illustrate the relationship of these two polymorphisms (rs13040307 and rs73115010) in SLE and RA.

For another two polymorphisms (rs1883832 and rs4810485), several studies have discussed the association with SLE, RA risk. Rs1883832 was significantly associated with SLE in a Tunisian population (22). However, a study in Egypt population and a study in Korea population did not find significant results (12, 23). For RA, there was no association of rs1883832 in Eastern Chinese and Iran population (24, 25) and the study published by Liu et al. showed that genotype TT versus CC+CT was significant in men (24). On the contrary, rs1883832 was related to RA risk in a Tunisian population (22). According to rs4810485, this polymorphism was confirmed related to SLE and RA risk in European population (11), but not in Korea and Tunisian population (12, 22). In the present study for Southern Chinese Han population, rs1883832 and rs4810485 was related to RA risk, whereas these two polymorphisms were not related to SLE by analyzing genetic models. However, these two polymorphisms were related to SLE patients complicated with myositis. Compared our findings with Liu et al., the differences may correlate with several reasons. First, Liu et al. genotyped the polymorphism by MALDI-TOF MS using the MassARRAY Nanodispenser, and we used KASP method. Second, we have larger sample size not only in RA patients but in healthy controls. Third, the author found a significant result regarding genotype TT versus CC+CT in men, while in our study, we did not find a significant result regarding genotype TT versus CC+CT in men (OR, 1.650; 95% CI, 0.651–4.180;P = 0.288), suggesting that whether this polymorphism may relate to RA risk either in all RA patients or male patients needs to be further discussed with larger sample size especially with larger men patients, the same genotyping method. Collectively, all these differences for SLE, RA risk as compared to our findings may relate to several possibilities. First, RA, SLE are complex diseases, and they have different pathogenesis, therefore, different polymorphisms may have distinct relation to SLE, RA risk, and it is possible that the same polymorphism may have different relation to SLE, RA. Second, different studies selected different methods to detect the polymorphisms may have distinct results. Third, different ethnicities may associate with different results.

Since CD40 gene polymorphisms may correlate with RA, SLE susceptibility, it is convinced that clarification of how polymorphism or what polymorphism of CD40 gene has affected mRNA expression and protein expression, and how does the protein perform in the pathogenesis of these diseases is meaningful and helpful. It is widely accepted that mRNA may be regulated by SNPs (26). Therefore, dysregulated expression of CD40 may be caused by upregulation of transcription and translation owing to mutated polymorphism. Studies showed that genotype TT, TC of rs1883832 associated with increased CD40 expression in Chinese and Egypt SLE patients (9, 23). Genotype GG of rs4810485 was related to increased CD40 expression in Greek and Turkish SLE patients (27, 28). CD40 is expressed on smooth muscle fibroblasts and synovial cells, which can be upregulated by pro-inflammatory cytokines, such as IFN-γ and TNFα. In turn, elevated expression of CD40 may further involve in the inflammatory response, leading to fibroblast proliferation, adhesion molecule upregulation, and generation of pro-inflammatory cytokines and chemokines, and finally causing diseases development and progression (7, 29). To date, several studies have showed that expression of CD40 was elevated in SLE, RA patients and associated with diseases activity (30–32). Therefore, mutation in CD40 gene may have affected CD40 expression, and promoted SLE, RA development. In our study, we did not discuss association of these polymorphism and CD40 expression in RA patients or SLE patients. Functional study in the future is necessary to elucidate how polymorphism regulates CD40 expression, especially the role in RA, SLE pathogenesis.

There are some limitations in the present study. First, selection bias cannot be avoided because patients were selected from a hospital. Second, this study focuses on association of SNPs with susceptibility to SLE, RA, however, exploring the process of transcription and translation of CD40 gene by the polymorphisms is of importance. Third, more SNPs of CD40 gene are needed to discuss association with SLE, RA risk, and discussion of gene-environment interaction should be investigated in the future. Fourth, we found association of myositis with the six polymorphisms, however, the conclusion is to take with caution because of the very low number of patients. Finally, replication of the results on other ethnics is lacking.

In conclusion, our study showed that CD40 gene polymorphisms may relate to SLE and RA susceptibility.

## Data Availability Statement

The raw data supporting the conclusions of this article will be made available by the authors, without undue reservation.

## Ethics Statement

The studies involving human participants were reviewed and approved by Ethic Research Committee of Southwest Medical University. The patients/participants provided their written informed consent to participate in this study.

## Author Contributions

QH, W-DX, L-CS, X-YL, and A-FH designed and wrote this paper. All authors contributed to the article and approved the submitted version.

## Funding

This work was supported by grants from the National Natural Science Foundation of China (81701606) and the Sichuan Provincial Science and Technology Program (2019YJ0540).

## Conflict of Interest

The authors declare that the research was conducted in the absence of any commercial or financial relationships that could be construed as a potential conflict of interest.
