# Supplementary material for: Association of CD40 Gene Polymorphisms With Systemic Lupus Erythematosus and Rheumatoid Arthritis in a Chinese Han Population
Source: Front Immunol. 2021 Apr 22;12:642929. doi: 10.3389/fimmu.2021.642929 (PMC8100582; doi:10.3389/fimmu.2021.642929)
Supplement: Supplementary file 1 [file Table_1.docx]

Supplementary Table 1 The primer information for CD40 gene polymorphisms

| ID | Primer AlleleFAM | Primer AlleleHEX | Primer Common | AlleleFAM | AlleleHEX |
| --- | --- | --- | --- | --- | --- |
| rs1569723 | ACTCGCTTTACACCCACAGCCA | CTCGCTTTACACCCACAGCCC | ATGGGCAAGGAAGAGGAAATTGGAATTTT | A | C |
| rs1883832 | CGCCTGGTCTCACCTCGCT | GCCTGGTCTCACCTCGCC | CACTGCAGAGGCAGACGAACCAT | T | C |
| rs4810485 | ACTTTAGAGGGCTGTAGATTCCG | CTACTTTAGAGGGCTGTAGATTCCT | TGGGTCATTCCTGCCCAGGCTT | G | T |
| rs3765456 | GTGGTCTCCCCTCCCTCCT | GGTCTCCCCTCCCTCCC | AGTCCTCAGGTGGGGAGGTGTT | A | G |
| rs13040307 | ATGTCTCCCAATCAGACCATCC | GTATGTCTCCCAATCAGACCATCT | GACCCTGCCTGCTAGGAGGAAA | C | T |
| rs73115010 | ATTTGTTAACTGTCTGCCTTTCCCC | ATTTGTTAACTGTCTGCCTTTCCCT | AAAGGCCAAGTTTCTACATTTAAGGAATTT | C | T |
