# Supplementary material for: Association of CD40 Gene Polymorphisms With Systemic Lupus Erythematosus and Rheumatoid Arthritis in a Chinese Han Population
Source: Front Immunol. 2021 Apr 22;12:642929. doi: 10.3389/fimmu.2021.642929 (PMC8100582; doi:10.3389/fimmu.2021.642929)
Supplement: Supplementary file 2 [file Table_2.docx]

Supplementary Table 2 The Hardy-Weinberg's expectation test in patients and controls of six polymorphisms.

| Single nucleotide polymorphism | Systemic lupus erythematosus | | Rheumatoid arthritis | | | | Healthy controls | |
| --- | --- | --- | --- | --- | --- | --- | --- | --- |
|  | χ^2^ | P |  | χ^2^ | P |  | χ^2^ | P |
| rs1883832 | 0.174 | 0.917 |  | 0.887 | 0.642 |  | 1.158 | 0.560 |
| rs1569723 | 0.133 | 0.916 |  | 0.887 | 0.642 |  | 0.605 | 0.739 |
| rs4810485 | 0.217 | 0.897 |  | 1.305 | 0.521 |  | 0.769 | 0.681 |
| rs13040307 | 1.224 | 0.542 |  | 1.041 | 0.594 |  | 0.312 | 0.856 |
| rs3765456 | 0.675 | 0.714 |  | 0.586 | 0.746 |  | 0.009 | 0.995 |
| rs73115010 | 0.854 | 0.652 |  | 0.019 | 0.991 |  | 0.188 | 0.910 |
