# Supplementary material for: Association of CD40 Gene Polymorphisms With Systemic Lupus Erythematosus and Rheumatoid Arthritis in a Chinese Han Population
Source: Front Immunol. 2021 Apr 22;12:642929. doi: 10.3389/fimmu.2021.642929 (PMC8100582; doi:10.3389/fimmu.2021.642929)
Supplement: Supplementary file 3 [file Table_3.docx]

Supplementary Table 3 Analysis of CD40 gene polymorphisms (rs1883832, rs1569723, rs4810485) in SLE patients by clinical features.

| Clinical features | rs1883832 | | | | | | | |  | rs1569723 | | | | | | | |  | rs4810485 | | | | | | | |
| --- | --- | --- | --- | --- | --- | --- | --- | --- | --- | --- | --- | --- | --- | --- | --- | --- | --- | --- | --- | --- | --- | --- | --- | --- | --- | --- |
|  | Genotype frequency (n) | | |  |  | Allele frequency (n) | |  |  | Genotype frequency (n) | | |  |  | Allele frequency (n) | |  |  | Genotype frequency (n) | | |  |  | Allele frequency (n) | |  |
|  |  |  |  |  |  |  |  |  |  |  | | |  |  |  |  |  |  |  |  |  |  |  |  |  |  |
|  | TT | TC | CC | P_1_ |  | T | C | P_2_ |  | AA | AC | CC | P_1_ |  | A | C | P_2_ |  | TT | TG | GG | P_1_ |  | T | G | P_2_ |
| Arthritis |  |  |  |  |  |  |  |  |  |  |  |  |  |  |  |  |  |  |  |  |  |  |  |  |  |  |
| Positive | 15 | 42 | 35 | 0.540 |  | 72 | 112 | 0.743 |  | 34 | 42 | 16 | 0.555 |  | 110 | 74 | 0.769 |  | 15 | 43 | 34 | 0.632 |  | 73 | 111 | 0.828 |
| Negative | 15 | 58 | 35 |  |  | 88 | 128 |  |  | 34 | 58 | 16 |  |  | 126 | 90 |  |  | 15 | 58 | 35 |  |  | 88 | 128 |  |
| Discoid rash |  |  |  |  |  |  |  |  |  |  |  |  |  |  |  |  |  |  |  |  |  |  |  |  |  |  |
| Positive | 13 | 41 | 29 | 0.980 |  | 67 | 99 | 0.901 |  | 26 | 44 | 13 | 0.750 |  | 96 | 70 | 0.689 |  | 13 | 41 | 29 | 0.962 |  | 67 | 99 | 0.969 |
| Negative | 17 | 59 | 41 |  |  | 93 | 141 |  |  | 42 | 56 | 19 |  |  | 140 | 94 |  |  | 17 | 60 | 40 |  |  | 94 | 140 |  |
| Alopecia |  |  |  |  |  |  |  |  |  |  |  |  |  |  |  |  |  |  |  |  |  |  |  |  |  |  |
| Positive | 9 | 24 | 17 | 0.815 |  | 42 | 58 | 0.637 |  | 16 | 25 | 9 | 0.887 |  | 57 | 43 | 0.639 |  | 9 | 25 | 16 | 0.802 |  | 43 | 57 | 0.517 |
| Negative | 21 | 76 | 53 |  |  | 118 | 182 |  |  | 52 | 75 | 23 |  |  | 179 | 121 |  |  | 21 | 76 | 53 |  |  | 118 | 182 |  |
| Oral ulcers |  |  |  |  |  |  |  |  |  |  |  |  |  |  |  |  |  |  |  |  |  |  |  |  |  |  |
| Positive | 5 | 10 | 9 | 0.615 |  | 20 | 28 | 0.802 |  | 7 | 12 | 5 | 0.769 |  | 26 | 22 | 0.468 |  | 5 | 10 | 9 | 0.589 |  | 20 | 28 | 0.831 |
| Negative | 25 | 90 | 61 |  |  | 140 | 212 |  |  | 61 | 88 | 27 |  |  | 210 | 142 |  |  | 25 | 91 | 60 |  |  | 141 | 211 |  |
| Pleurisy |  |  |  |  |  |  |  |  |  |  |  |  |  |  |  |  |  |  |  |  |  |  |  |  |  |  |
| Positive | 3 | 7 | 7 | 0.788 |  | 13 | 21 | 0.826 |  | 6 | 8 | 3 | 1.000 |  | 20 | 14 | 0.983 |  | 3 | 7 | 7 | 0.738 |  | 13 | 21 | 0.802 |
| Negative | 27 | 93 | 63 |  |  | 147 | 219 |  |  | 62 | 92 | 29 |  |  | 216 | 150 |  |  | 27 | 94 | 62 |  |  | 148 | 218 |  |
| Pericarditis |  |  |  |  |  |  |  |  |  |  |  |  |  |  |  |  |  |  |  |  |  |  |  |  |  |  |
| Positive | 2 | 5 | 9 | 0.205 |  | 9 | 23 | 0.153 |  | 7 | 7 | 2 | 0.733 |  | 21 | 11 | 0.427 |  | 2 | 6 | 8 | 0.407 |  | 10 | 22 | 0.279 |
| Negative | 28 | 95 | 61 |  |  | 151 | 217 |  |  | 61 | 93 | 30 |  |  | 215 | 153 |  |  | 28 | 95 | 61 |  |  | 151 | 217 |  |
| Fever |  |  |  |  |  |  |  |  |  |  |  |  |  |  |  |  |  |  |  |  |  |  |  |  |  |  |
| Positive | 7 | 18 | 13 | 0.853 |  | 32 | 44 | 0.677 |  | 14 | 17 | 7 | 0.812 |  | 45 | 31 | 0.967 |  | 7 | 18 | 13 | 0.836 |  | 32 | 44 | 0.714 |
| Negative | 23 | 82 | 57 |  |  | 128 | 196 |  |  | 54 | 83 | 25 |  |  | 191 | 133 |  |  | 23 | 83 | 56 |  |  | 129 | 195 |  |
| Hypocomplementemia | |  |  |  |  |  |  |  |  |  |  |  |  |  |  |  |  |  |  |  |  |  |  |  |  |  |
| Positive | 20 | 44 | 36 | 0.086 |  | 84 | 116 | 0.414 |  | 34 | 44 | 22 | 0.055 |  | 112 | 88 | 0.222 |  | 20 | 45 | 35 | 0.105 |  | 85 | 115 | 0.359 |
| Negative | 10 | 56 | 34 |  |  | 76 | 124 |  |  | 34 | 56 | 10 |  |  | 124 | 76 |  |  | 10 | 56 | 34 |  |  | 76 | 124 |  |
| ds-DNA(+) |  |  |  |  |  |  |  |  |  |  |  |  |  |  |  |  |  |  |  |  |  |  |  |  |  |  |
| Positive | 10 | 26 | 12 | 0.186 |  | 46 | 50 | 0.069 |  | 14 | 23 | 11 | 0.327 |  | 51 | 45 | 0.179 |  | 10 | 26 | 12 | 0.204 |  | 46 | 50 | 0.079 |
| Negative | 20 | 74 | 58 |  |  | 114 | 50 |  |  | 54 | 77 | 21 |  |  | 185 | 119 |  |  | 20 | 75 | 57 |  |  | 115 | 189 |  |
| Thrombocytopenia | |  |  |  |  |  |  |  |  |  |  |  |  |  |  |  |  |  |  |  |  |  |  |  |  |  |
| Positive | 5 | 14 | 10 | 0.964 |  | 24 | 34 | 0.817 |  | 10 | 14 | 5 | 1.000 |  | 34 | 24 | 0.949 |  | 5 | 14 | 10 | 0.964 |  | 24 | 34 | 0.850 |
| Negative | 25 | 86 | 60 |  |  | 136 | 206 |  |  | 58 | 86 | 27 |  |  | 202 | 140 |  |  | 25 | 87 | 59 |  |  | 137 | 205 |  |
| Leukopenia |  |  |  |  |  |  |  |  |  |  |  |  |  |  |  |  |  |  |  |  |  |  |  |  |  |  |
| Positive | 6 | 10 | 8 | 0.340 |  | 22 | 26 | 0.379 |  | 8 | 10 | 6 | 0.415 |  | 26 | 22 | 0.468 |  | 6 | 10 | 8 | 0.353 |  | 22 | 26 | 0.400 |
| Negative | 24 | 90 | 62 |  |  | 138 | 214 |  |  | 60 | 90 | 26 |  |  | 210 | 142 |  |  | 24 | 91 | 61 |  |  | 139 | 213 |  |
| Hematuria |  |  |  |  |  |  |  |  |  |  |  |  |  |  |  |  |  |  |  |  |  |  |  |  |  |  |
| Positive | 15 | 29 | 21 | 0.093 |  | 59 | 71 | 0.127 |  | 19 | 31 | 15 | 0.165 |  | 69 | 61 | 0.095 |  | 15 | 30 | 20 | 0.089 |  | 60 | 70 | 0.095 |
| Negative | 15 | 71 | 49 |  |  | 101 | 169 |  |  | 49 | 69 | 17 |  |  | 167 | 103 |  |  | 15 | 71 | 49 |  |  | 101 | 169 |  |
| Proteinuria |  |  |  |  |  |  |  |  |  |  |  |  |  |  |  |  |  |  |  |  |  |  |  |  |  |  |
| Positive | 17 | 44 | 35 | 0.458 |  | 78 | 114 | 0.806 |  | 33 | 44 | 19 | 0.311 |  | 110 | 82 | 0.504 |  | 17 | 45 | 34 | 0.502 |  | 79 | 113 | 0.760 |
| Negative | 13 | 56 | 35 |  |  | 82 | 126 |  |  | 35 | 56 | 13 |  |  | 126 | 82 |  |  | 13 | 56 | 35 |  |  | 82 | 126 |  |
| Pyuria |  |  |  |  |  |  |  |  |  |  |  |  |  |  |  |  |  |  |  |  |  |  |  |  |  |  |
| Positive | 6 | 9 | 7 | 0.243 |  | 21 | 23 | 0.328 |  | 6 | 10 | 6 | 0.350 |  | 22 | 22 | 0.198 |  | 6 | 9 | 7 | 0.225 |  | 21 | 23 | 0.284 |
| Negative | 24 | 91 | 63 |  |  | 139 | 217 |  |  | 62 | 90 | 26 |  |  | 214 | 142 |  |  | 24 | 92 | 63 |  |  | 140 | 216 |  |
| Vasculitis |  |  |  |  |  |  |  |  |  |  |  |  |  |  |  |  |  |  |  |  |  |  |  |  |  |  |
| Positive | 2 | 12 | 4 | 0.329 |  | 16 | 20 | 0.568 |  | 4 | 12 | 2 | 0.333 |  | 20 | 16 | 0.660 |  | 2 | 12 | 4 | 0.352 |  | 16 | 20 | 0.591 |
| Negative | 28 | 88 | 66 |  |  | 144 | 220 |  |  | 64 | 88 | 30 |  |  | 216 | 148 |  |  | 28 | 89 | 65 |  |  | 145 | 219 |  |
| Myositis |  |  |  |  |  |  |  |  |  |  |  |  |  |  |  |  |  |  |  |  |  |  |  |  |  |  |
| Positive | 0 | 4 | 7 | 0.086 |  | 4 | 18 | 0.032 |  | 7 | 4 | 0 | 0.071 |  | 18 | 4 | 0.020 |  | 0 | 4 | 7 | 0.079 |  | 18 | 4 | 0.030 |
| Negative | 30 | 96 | 63 |  |  | 156 | 222 |  |  | 61 | 96 | 32 |  |  | 218 | 160 |  |  | 30 | 97 | 62 |  |  | 218 | 160 |  |

P_1_: Patients positive versus patients negative using 3×2 contingency table.

P_2_: Patients positive versus patients negative using 2×2 contingency table.
