# Supplementary material for: Association of CD40 Gene Polymorphisms With Systemic Lupus Erythematosus and Rheumatoid Arthritis in a Chinese Han Population
Source: Front Immunol. 2021 Apr 22;12:642929. doi: 10.3389/fimmu.2021.642929 (PMC8100582; doi:10.3389/fimmu.2021.642929)
Supplement: Supplementary file 4 [file Table_4.docx]

| Locus | Genetic model | Gene types | SLEDAI  [Median (P_25_-P_75_)] | P |  | Locus | Genetic model | Gene types | SLEDAI  [Median (P_25_-P_75_)] | P |
| --- | --- | --- | --- | --- | --- | --- | --- | --- | --- | --- |
| rs1883832  (C > T) | Co-dominant model | CC | 10.00 (5.75-12.25) | 0.521 |  | rs13040307  (C>T) | Co-dominant model | CC | 10.00 (4.00-15.0) | 0.742 |
|  |  | CT | 8.00 (4.00-13.00) | 0.122 |  |  |  | CT | 8.00 (4.00-12.00) | 0.154 |
|  |  | TT | 10.50 (4.00-18.50) | - |  |  |  | TT | 10.50 (6.00-12.00) | - |
|  | Recessive model | CT+TT | 8.00 (4.00-13.25) | 0.344 |  |  | Recessive model | CT+TT | 8.00 (4.00-12.00) | 0.344 |
|  | Dominant model | CT+CC | 8.50 (4.00-13.00) | 0.211 |  |  | Dominant model | CT+CC | 8.50 (4.00-13.00) | 0.370 |
|  |  |  |  |  |  |  |  |  |  |  |
| rs1569723 (A>C) | Co-dominant model | AA | 9.50 (5.00-12.00) | 0.576 |  | rs3765456  (G>A) | Co-dominant model | GG | 10.50 (6.00-13.00) | 0.060 |
|  |  | AC | 8.00 (4.00-13.00) | 0.232 |  |  |  | GA | 8.00 (4.00-13.00) | 0.258 |
|  |  | CC | 9.50 (4.00-17.50) | - |  |  |  | AA | 8.00 (4.00-16.00) | - |
|  | Recessive model | AC+CC | 8.00 (4.00-14.00) | 0.615 |  |  | Recessive model | GA+AA | 8.00 (4.00-13.00) | 0.136 |
|  | Dominant model | AC+AA | 9.00 (4.00-13.00) | 0.316 |  |  | Dominant model | GA+GG | 9.00 (4.00-13.00) | 0.565 |
|  |  |  |  |  |  |  |  |  |  |  |
| rs4810485  (G>T) | Co-dominant model | GG | 10.00 (5.50-12.00) | 0.496 |  | rs73115010  (T>C) | Co-dominant model | TT | 10.00 (4.00-15.00) | 0.282 |
|  |  | GT | 8.00 (4.00-13.00) | 0.131 |  |  |  | TC | 8.00 (4.00-13.00) | 0.313 |
|  |  | TT | 10.50 (4.00-18.50) | - |  |  |  | CC | 10.00 (5.50-12.00) |  |
|  | Recessive model | GT+TT | 8.00 (4.00-14.00) | 0.419 |  |  | Recessive model | TC+CC | 8.00 (4.00-12.00) | 0.437 |
|  | Dominant model | GT+ GG | 8.50 (4.00-13.00) | 0.211 |  |  | Dominant model | TC+TT | 9.00 (4.00-14.00) | 0.528 |

Supplementary Table 4 Systemic Lupus Erythematosus Disease activity index (SLEDAI) in relation to CD40 gene polymorphisms.
