# Supplementary material for: Association of CD40 Gene Polymorphisms With Systemic Lupus Erythematosus and Rheumatoid Arthritis in a Chinese Han Population
Source: Front Immunol. 2021 Apr 22;12:642929. doi: 10.3389/fimmu.2021.642929 (PMC8100582; doi:10.3389/fimmu.2021.642929)
Supplement: Supplementary file 5 [file Table_5.docx]

| Characteristics | rs4810485 [Median (P_25_-P_75_)] | | |  |  | rs1569723 [Median (P_25_-P_75_)] | | | |
| --- | --- | --- | --- | --- | --- | --- | --- | --- | --- |
|  | GG | TG | TT | P_1_ |  | AA | AC | CC | P_2_ |
| Tender joints (n) | 10.00 (4.00-24.50) | 12.00 (5.00-24.00) | 12.00 (4.00-26.00) | 0.726 |  | 12.00 (3.00-23.50) | 11.00 (5.00-27.00) | 12.0 (4.00-24.00) | 0.727 |
| Swollen joints (n) | 6.00 (1.00-14.00) | 8.00 (2.00-16.00) | 6.00 (2.00-17.00) | 0.930 |  | 4.00 (0.00-12.00) | 10.00 (2.00-20.00) | 7.00 (2.00-16.00) | 0.498 |
| ESR (n) | 52.00 (27.75-98.00) | 61.00 (35.00-90.00) | 68.00 (40.50-95.00) | 0.407 |  | 55.50 (26.50-100.50) | 60.00 (31.5-87.00) | 61.00 (36.00-95.00) | 0.761 |
| CRP (mg/L) | 17.80 (4.80-50.30) | 24.70 (5.13-63.40) | 18.80 (7.20-49.50) | 0.671 |  | 26.00 (6.40-54.35) | 19.90 (4.21-50.00) | 21.05 (5.50-58.00) | 0.479 |
| Self-evaluation | 75.00 (65.00-85.00) | 70.00 (65.00-80.00) | 78.00 (70.00-81.50) | 0.277 |  | 70.00 (62.50-85.00) | 75.00 (65.00-85.00) | 72.50 (66.50-80.00) | 0.300 |
| HAQ score | 20.00 (12.00-27.00) | 20.00 (9.00-27.25) | 20.50 (7.00-28.50) | 0.991 |  | 20.00 (11.50-26.50) | 20.00 (8.25-27.00) | 20.00 (10.00-28.00) | 0.458 |
| IgG (g/L) | 13.18 (9.58-14.40) | 12.95 (9.41-15.73) | 13.07 (11.43-18.15) | 0.381 |  | 13.34 (9.53-15.71) | 12.95 (9.84-16.40) | 13.04 (9.31-16.30) | 0.688 |
| IgA (mg/L) | 2.38 (1.80-3.12) | 2.26 (1.74-3.12) | 2.79 (2.23-4.53) | 0.051 |  | 1.98 (1.74-2.75) | 2.40 (1.79-3.01) | 2.34 (1.67-3.64) | 0.140 |
| IgM (mg/L) | 1.22 (0.89-2.01) | 1.34 (0.96.00-2.12) | 1.48 (1.26-2.05) | 0.325 |  | 1.51 (0.80-2.07) | 1.27 (0.96-2.09) | 1.36 (0.99-1.95) | 0.632 |
| RF (IU/L) | 138.55 (40.50-232.75) | 122.65 (33.60-293.83) | 112.90 (30.80-203.0) | 0.573 |  | 91.45 (34.98-268.78) | 131.35 (36.67-232.25) | 129.10 (38.15-258.95) | 0.540 |
| anti-CCP (U/mL) | 63.00 (28.60-124.00) | 57.80 (30.60-116.90) | 73.00 (25.40-110.70) | 0.932 |  | 63.00 (27.2-99.00) | 58.40 (31.50-148.15) | 58.75 (25.93-111.38) | 0.577 |
| C3 (g/L) | 1.28 (1.16-1.38) | 1.22 (1.08-1.44) | 1.92 (1.12-1.46) | 0.594 |  | 1.28 (1.16-1.43) | 1.23 (1.12-1.37) | 1.28 (1.10-1.46) | 0.718 |
| C4 (g/L) | 0.25 (0.21-0.36) | 0.28 (0.22-0.32) | 0.29 (0.24-0.41) | 0.182 |  | 0.29 (0.19-0.37) | 0.27 (0.22-0.31) | 0.26 (0.21-0.34) | 0.330 |
| DAS28 | 6.14 (4.96-7.53) | 6.40 (5.48-7.46) | 6.78 (5.16-7.94) | 0.414 |  | 6.14 (5.00-7.52) | 6.49 (5.47-7.54) | 6.70 (5.16-7.57) | 0.963 |

Supplementary Table 5 Disease activity parameters of RA patients in relation to CD40 gene polymorphisms (rs4810485, rs1569723).

ESR: erythrocyte sedimentation rate; CRP: C-reactive protein; HAQ: health assessment questionnaire; RF: rheumatoid factor; DAS28, disease activity score 28.

^1^GG+TG versus TT genotype for rs4810485; ^2^AA+AC versus CC genotype for rs1569723
