# Supplementary material for: Association of CD40 Gene Polymorphisms With Systemic Lupus Erythematosus and Rheumatoid Arthritis in a Chinese Han Population
Source: Front Immunol. 2021 Apr 22;12:642929. doi: 10.3389/fimmu.2021.642929 (PMC8100582; doi:10.3389/fimmu.2021.642929)
Supplement: Supplementary file 6 [file Table_6.docx]

Supplementary Table 6 Disease activity parameters of RA patients in relation to CD40 gene polymorphisms (rs3765456, rs1883832).

| Characteristics | rs3765456 [Median (P_25_-P_75_)] | | |  |  | rs1883832 [Median (P_25_-P_75_)] | | |  |
| --- | --- | --- | --- | --- | --- | --- | --- | --- | --- |
|  | AA | AG | GG | P_1_ |  | TT | TC | CC | P_2_ |
| Tender joints (n) | 10.00 (4.00-23.00) | 14.00 (5.00-24.00) | 10.00 (4.00-26.00) | 0.449 |  | 12.00 (4.00-26.00) | 12.00 (5.00-23.75) | 10.00 (4.00-25.75) | 0.726 |
| Swollen joints (n) | 6.00 (1.00-12.00) | 10.00 (2.25-20.00) | 5.00 (1.00-14.00) | 0.173 |  | 9.00 (2.00-16.00) | 8.00 (2.00-16.00) | 6.00 (1.00-14.00) | 0.909 |
| ESR (n) | 70.00 (37.00-96.60) | 61.00 (38.00-87.80) | 53.00 (27.50-98.00) | 0.381 |  | 65.50 (40.50-91.75) | 61.50 (35.0-91.25) | 51.00 (27.50-97.00) | 0.608 |
| CRP (mg/L) | 16.43 (6.00-55.40) | 24.70 (5.10-59.70) | 17.90 (4.78-51.30) | 0.648 |  | 18.80 (7.20-41.25) | 26.20 (5.15-64.40) | 17.70 (4.78-49.63) | 0.394 |
| Self-evaluation | 75.00 (65.00-80.00) | 70.00 (65.00-80.00) | 75.00 (65.00-85.00) | 0.805 |  | 80.00 (70.00-84.00) | 70.00 (65.00-80.00) | 75.00 (65.00-85.00) | 0.210 |
| HAQ score | 19.50 (7.00-26.50) | 20.00 (10.00-29.00) | 20.00 (12.00-27.00) | 0.292 |  | 21.00 (7.00-30.00) | 20.00 (9.00-27.00) | 20.00 (12.00-27.00) | 0.812 |
| IgG (g/L) | 13.98 (11.16-19.49) | 13.01 (9.19-15.74) | 12.16 (9.60-16.00) | 0.139 |  | 11.91 (11.11-17.65) | 13.87 (9.43-16.06) | 12.57 (9.58-16.27) | 0.776 |
| IgA (mg/L) | 2.78 (1.86-4.37) | 2.31 (1.77-3.13) | 2.30 (1.77-2.97) | 0.102 |  | 2.69 (1.95-4.37) | 2.28 (1.76-3.13) | 2.35 (1.78-3.05) | 0.167 |
| IgM (mg/L) | 1.51 (1.09-2.08) | 1.30 (0.95-2.04) | 1.20 (0.90-1.98) | 0.221 |  | 1.55 (1.27-2.09) | 1.33 (0.96-1.96) | 1.22 (0.91-2.04) | 0.171 |
| RF (IU/L) | 138.55 (62.15-237.00) | 118.60 (23.85-293.95) | 134.80 (41.38-226.25) | 0.479 |  | 112.90 (30.80-222.00) | 125.50 (33.00-294.20) | 138.10 (40.30-232.00) | 0.411 |
| anti-CCP (U/mL) | 59.00 (29.75-98.50) | 65.70 (31.00-122.25) | 53.20 (27.28-124.13) | 0.826 |  | 79.65 (25.47-241.43) | 56.55 (29.40-112.55) | 63.00 (29.60-125.85) | 0.746 |
| C3 (g/L) | 1.21 (1.10-1.46) | 1.26 (1.09-1.44) | 1.28 (1.14-1.38) | 0.786 |  | 1.33 (1.13-1.46) | 1.21 (1.08-1.44) | 1.29 (1.16-1.41) | 0.527 |
| C4 (g/L) | 0.26 (0.21-0.36) | 0.28 (0.23-0.32) | 0.26 (0.21-0.36) | 0.740 |  | 0.30 (0.23-0.39) | 0.27 (0.22-0.32) | 0.26 (0.21-0.36) | 0.253 |
| DAS28 | 6.65 (5.37-7.45) | 6.49 (5.40-7.63) | 6.17 (5.14-7.51) | 0.892 |  | 6.76 (5.02-7.93) | 6.39 (5.48-7.46) | 6.17 (5.00-7.55) | 0.543 |

ESR: erythrocyte sedimentation rate; CRP: C-reactive protein; HAQ: health assessment questionnaire; RF: rheumatoid factor; DAS28, disease activity score 28.

^1^AA+AG versus GG genotype for rs3765456; ^2^TT+TC versus CC genotype for rs1883832.
