# Supplementary material for: Association of CD40 Gene Polymorphisms With Systemic Lupus Erythematosus and Rheumatoid Arthritis in a Chinese Han Population
Source: Front Immunol. 2021 Apr 22;12:642929. doi: 10.3389/fimmu.2021.642929 (PMC8100582; doi:10.3389/fimmu.2021.642929)
Supplement: Supplementary file 7 [file Table_7.docx]

Supplementary Table 7 Haplotype analysis for patients and controls.

| Blocks | Haplotype | SLE ratio | RA ratio | Control ratio | χ^2^_a_ | P _a_ |  | χ^2^_b_ | P _b_ |
| --- | --- | --- | --- | --- | --- | --- | --- | --- | --- |
| 1 | CCTT | 0.392 | 0.397 | 0.397 | 1.078 | 0.299 |  | 0.038 | 0.846 |
|  | ATCG | 0.330 | 0.298 | 0.298 | 0.027 | 0.868 |  | 1.660 | 0.198 |
|  | ACCG | 0.257 | 0.308 | 0.281 | 1.753 | 0.186 |  | 0.922 | 0.337 |
| 2 | TA | 0.417 | 0.397 | 0.413 | 0.541 | 0.462 |  | 0.023 | 0.878 |
|  | CG | 0.350 | 0.326 | 0.318 | 0.171 | 0.679 |  | 1.552 | 0.213 |
|  | TG | 0.233 | 0.276 | 0.269 | 0.144 | 0.705 |  | 2.240 | 0.135 |

RA: rheumatoid arthritis; SLE: systemic lupus erythematosus.

^a^RA patient versus controls using 2×2 chi-square test; ^b^SLE patient versus controls using 2×2 chi-square test.
